# Impact of lowering the maximum speed limit of city roads on pedestrian traffic accident patients: A nationwide before-and-after study

**DOI:** 10.1371/journal.pone.0325320

**Published:** 2025-06-11

**Authors:** Hun Gi Lee, Jung Ho Kim

**Affiliations:** Department of Emergency Medicine, Yeungnam University College of Medicine, Daegu, South Korea; Jazan University College of Applied Medical Science, SAUDI ARABIA

## Abstract

Traffic accidents (TAs) remain the leading cause of death globally; therefore, numerous efforts have been made to improve their occurrence and outcomes. Lowering the maximum road speed limit, named Safe Speed-5030, implemented in South Korea on April 17, 2021, is an example. This before-and-after study investigated the impact of this policy on pedestrian TA (P-TA) patients. We used the National Emergency Department Information System data for seven major cities in South Korea. We compared the characteristics of the patients who underwent P-TA and assessed the risks of emergency surgery, intensive care unit (ICU) admission, and unfavorable outcomes, using IBM SPSS version 21.0. The total number of patients who met with P-TAs was 26,842, with a 43.1% reduction from 17,105–9,737. A decrease was observed across all age groups; however, the rate was lower in the geriatric and severely injured groups. The proportions of emergency surgeries (46.5%), ICU admissions (36.5%), and unfavorable outcomes (21.5%) decreased. Nevertheless, this decrease was less pronounced in geriatric patients, and there was an increase in unfavorable outcomes among those in their 70s. After adjustment, the risk of emergency surgery did not show a statistically significant difference, but the risk of ICU admission and unfavorable outcomes increased. In conclusion, there was a significant reduction in the number of P-TA patients after the policy was implemented, but the effect was less pronounced in severely injured and geriatric patients. Considering these factors, we believe that Safe Speed-5030 had partly positive effects. Applying a suitably revised policy for specific group rather than completely scrapping it would be more appropriate.

## Introduction

Despite advancements in medical science, traffic accidents (TAs) remain the leading cause of death worldwide. The number of deaths caused by TAs worldwide has increased from 1.15 million in 2000 to 1.19 million in 2021 and remains the leading cause of death among individuals aged 5–29 years, as well as the 12th leading cause of death across all age groups [1].

Pedestrians are one of the most vulnerable targets in TAs. There were previous studies about pedestrians` behavior on the road. BakhtariAghdam et al. reported that risky behaviors were more frequently observed among male and young adult pedestrians, and emphasized the need for education using diverse methods and environmental interventions [2]. Aghabayk et al. reported that males were more likely to cross during a flashing signal, elderly individuals were less cautious at unsignalized crosswalks, and pedestrians using their phones exhibited the least cautious behaviors [3]. Xing et al. reported that factors such as crossing distance, traffic volume, red-light duration, number of lanes, and crosswalk type all contribute to pedestrian violations, with the installation of waiting refuge islands having the most significant impact [4]. The risks associated with texting and web-surfing on a phone while walking, as well as the need for various measures and strategies, are already well known [5]. However, control the pedestrians` behavior and reducing risk of the major injury from TAs still challenging. Numerous efforts have been made to overcome this situation, one of which is to control vehicle speed. Previous studies have reported the relationship between vehicle speed and TA. Rossi et al. reported that a reduction in the speed limit of 30 km/h in urban areas is associated with reduced TAs and health benefits from decreased noise exposure [6]. Graham et al. reported that vehicle collisions significantly decreased in speed camera sites [7]. Lawrence et al. stated that strengthening the speed limit in residential areas from 40 km/h to 30 km/h is acceptable to residents and reduces pedestrian injury risk by approximately 4% [8]. Guo et al. reported that rectangular markings on crosswalks reduced vehicle speeds and resulted in a 24.9% decrease in vehicle-pedestrian collisions [9]. However, according to Hunter et al., implementing a 20 mph speed limit in urban areas had little effect on TAs or casualties, except for a decrease in traffic volume, indicating that controversy remains [10].

In South Korea, a policy named Safe-Speed-5030, which lowers the speed limit on urban roads to reduce TA occurrence and fatality rates, took effect on April 17, 2021, after a two-year grace period following legislation in April 2019. With this policy, the maximum speed limit on urban roads, excluding expressways, ring roads, and highways, was decreased from the previous limits [11]. Pedestrians are directly exposed to the external environment; in the event of a TA, they can sustain severe injuries and experience high fatality rates. Moreover, as roads excluded by this policy restrict pedestrian access by law, this policy likely has had a significant impact on pedestrian TAs (P-TAs). However, the Safe-Speed-5030 policy has stirred dissatisfaction and controversy among many citizens, leading to opinions about its abolition being voiced by local governments [12]. Nevertheless, if a policy is deemed to benefit the public despite social controversy, it should be implemented appropriately through consensus-building and promotion efforts based on improvements with scientific evidence. This study investigates the Safe-Speed-5030 policy’s impact on the incidence and outcomes of P-TA patients to provide insights to aid in decisions regarding policy modification or abolition.

## Methods

### Policy

Safe-Speed-5030 is a policy that lowers the speed limit on urban roads in South Korea. The purpose of this policy is to reduce the number of TA victims. This policy was implemented nationwide on April 17, 2021, after a two-year grace period. According to this policy, maximum urban road speeds were adjusted as follows: for urban general roads, from 60–80 km/h to 50–60 km/h; and for urban back roads, from 40–60 km/h to 30 km/h. The roads subject to the policy are those that are used by vehicles, motorcycles, bicycles, and pedestrians simultaneously. However, the existing speed limits are maintained for expressways, ring roads, highways, and rural roads [11].

### Study participants

This study involved P-TA patients who visited level-1 and level-2 emergency medical centers in seven major cities in South Korea. The cities included in the study were Seoul, Busan, Incheon, Daegu, Daejeon, Gwangju, and Ulsan. The overall population of these cities occupied 43.5% (22,544,933) of South Korea—9,708,247 in Seoul, 3,401,072 in Busan, 2,943,491 in Incheon, 2,428,228 in Daegu, 1,469,431 in Daejeon, 1,454,154 in Gwangju, and 1,140,310 in Ulsan [13]. The major emergency medical services in these cities were provided by 16 level-1 emergency medical centers—six in Seoul, one in Busan, two in Daegu, two in Incheon, two in Gwangju, two in Daejeon, and one in Ulsan—and 52 level-2 emergency medical centers—24 in Seoul, eight in Busan, four in Daegu, eight in Incheon, four in Gwangju, three in Daejeon, and one in Ulsan [14].

### Data collection

In South Korea, the National Emergency Medical Center (NEMC) operates an information collection system for patients admitted to major emergency medical centers nationwide known as the National Emergency Department Information System (NEDIS). Information is gathered in real time through the automatic transmission of electronic medical records from patients admitted to emergency medical centers. Quality control personnel at each emergency medical center and the NEMC verified the transmitted data to ensure accuracy. This study was conducted using customized data from the NEDIS (No 2022-11-01) on P-TA patients admitted to major emergency medical centers in the study cities. The NEDIS data cannot be publicly accessed and must be obtained by requesting it from the NEMC. It is provided after removing personally identifiable information and selecting relevant variables, following the NEMC’s review process and approval. The NEDIS data for this study was finally obtained from NEMC on March 6, 2024, and access for research purposes was then initiated.

This before-after study defined the period before policy implementation as one year from April 17, 2018, and the period after policy implementation as one year from April 17, 2021. We collected demographic data, including sex, age, region of visit to the ED, type of healthcare insurance, time of accident, level of visit to the ED, and arrival type at the ED. Furthermore, clinical data, including vital signs upon arrival at the ED, level of consciousness, the severity of P-TA patients, results of ED management, need for emergency surgery, and final treatment outcomes, were collected. Age was classified into seven categories: 20–29, 30–39, 40–49, 50–59, 60–69, 70–79, and over 80 years old. The time of the accidents was classified into four categories: 00:00–05:59 (dawn), 06:00–11:59 (morning), 12:00–17:59 (afternoon), and 18:00–23:59 (night). The severity of P-TA patients was categorized according to the Korean Triage and Acuity Scale (KTAS), a grading system used in emergency medical centers in South Korea since 2015 [15]. This system classifies patients into five levels, ranging from most critical to least critical—resuscitation, emergent, urgent, less urgent, and nonurgent—based on the patient’s severity. Unfavorable outcomes were defined as either death or discharge without the prospect of improvement.

### Statistical analysis

Categorical variables were presented as numbers and percentages, and Pearson’s chi-square test was performed. Moreover, univariable and multivariable logistic regression analyses were conducted for the association between policy implementation and the risk of emergency surgery, intensive care unit (ICU) admission, and unfavorable outcomes for identifying the impact of policy implementation. Confounding factors for the multivariable logistic regression analysis included sex, age, region of ED, type of healthcare insurance, time of accident, level of ED, arrival type at the ED, systolic blood pressure, heart rate, respiratory rate, body temperature, and level of consciousness. The results of the logistic regression analysis were presented as 95% confidence interval (CI), odds ratio (OR), and adjusted OR (aOR).

Statistical analysis was conducted using IBM SPSS version 21.0 (IBM Co., Armonk, NY, USA), and statistical significance was set as a p-value <0.05.

### Ethical considerations

This study was approved by the Institutional Review Board (IRB) of the Yeungnam University Hospital (No. 2022-04-050). All data were fully anonymized before being obtained from the NEMC, and the need for informed consent was waived by the IRB.

## Results

### Demographic characteristics

There were 26,842 P-TA patients, with 17,105 in the before-period and 9,737 in the after-period—a decrease of 7,368 (43.1%). The number of males was 12,870, with 8,360 in the before-period and 4,510 in the after-period—a decrease of 3,850 (46.1%). There was a decrease in the number of patients in all age groups; however, the rate was lower in the elderly (33.7% in their 60s, 36.7% in their 70s, and 18.8% in those older than 80). There was a decrease in the number of patients in all cities; nonetheless, Busan showed only an 11.0% rate decrease. At the time of TA occurrence, dawn exhibited the largest decrease at 62.3%. Table 1 presents the demographic characteristics of the participants.

**Table 1:** Demographic characteristics of the study population.

Variable	Total (n = 26,842)	Before (n = 17,105)	After (n = 9,737)	P-value
Sex				<0.001
Male	12,870 (47.9)	8,360 (48.9)	4,510 (46.3)	
Female	13,972 (52.1)	8,745 (51.1)	5,227 (53.7)	
Age (yr)				<0.001
20-29	4,366 (16.3)	2,860 (16.7)	1,506 (15.5)	
30-39	3,011 (11.2)	1,965 (11.5)	1,046 (10.7)	
40-49	3,345 (12.5)	2,268 (13.3)	1,077 (11.1)	
50-59	5,089 (19.0)	3,430 (20.1)	1,659 (17.0)	
60-69	5,000 (18.6)	3,007 (17.6)	1,993 (20.5)	
70-79	4,080 (15.2)	2,498 (14.6)	1,582 (16.2)	
≥ 80	1,951 (7.3)	1,077 (6.3)	874 (9.0)	
Region of hospital				<0.001
Seoul	14,246 (53.1)	9,340 (54.6)	4,906 (50.4)	
Busan	2,156 (8.0)	1,141 (6.7)	1,015 (10.4)	
Daegu	1,659 (6.2)	1,079 (6.3)	580 (6.0)	
Inchon	4,414 (16.4)	2,682 (15.7)	1,732 (17.8)	
Kwangju	1,345 (5.0)	856 (5.0)	489 (5.0)	
Daejeon	2,225 (8.3)	1,518 (8.9)	707 (7.3)	
Ulsan	797 (3.0)	489 (2.9)	308 (3.2)	
Health care insurance ^a^				<0.001
NHIS	3,857 (14.4)	2,164 (12.7)	1,693 (17.4)	
Auto insurance	21,872 (81.5)	14,237 (83.2)	7,635 (78.5)	
IACI	98 (0.4)	48 (0.3)	50 (0.5)	
Medicaid	412 (1.5)	262 (1.5)	150 (1.5)	
Others	597 (2.2)	394 (2.3)	203 (2.1)	
Time of accident ^b^				<0.001
Dawn	3,200 (11.9)	2,324 (13.6)	876 (9.0)	
Morning	6,197 (23.1)	3,874 (22.7)	2,323 (23.9)	
Afternoon	8,011 (29.9)	4,975 (29.1)	3,036 (31.2)	
Night	9,421 (35.1)	5,925 (22.1)	3,496 (35.9)	
Level of ED				<0.001
Level-1	7,057 (26.3)	4,766 (27.9)	2,291 (23.5)	
Level-2	19,785 (73.7)	12,339 (72.1)	7,446 (76.5)	

Presented as number (%)

^a^6 missing data point,

^b^13 missing data point. NHI, National Health Insurance Service; IACI, Industrial Accident Compensation Insurance; ED, emergency department; OPD, outpatient department.

### Clinical characteristics

**Fig 1:**
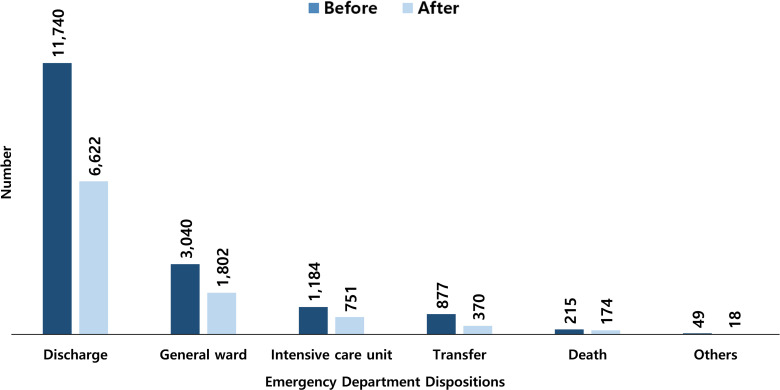
Emergency department dispositions of the study population.

P-TA patients with bradycardia showed a low rate of decrease (18.4%), and the proportion increased in the after-period from 2.6% to 3.8%. Additionally, those with systolic blood pressure less than 90 mmHg exhibited a low rate of decrease (29.5%), and the proportion increased in the after-period from 2.4% to 2.9%. There was no statistically significant difference in the level of consciousness upon ED arrival between the two periods (Table 2). The number of patients discharged from the ED decreased by 43.6%, from 11,740 in the before-period to 6,622 in the after-period, and the number of patients transferred to other hospitals also decreased by 57.8%, from 877 in the before-period to 370 in the after-period. However, the number of patients who died in the ED decreased by 19.1%, from 215 in the before-period to 174 in the after-period (Fig 1). The number of patients requiring emergency surgery decreased by 36.5% (165 patients) in the after-period, while ICU admission decreased by 36.6% (433 patients). The number of patients with unfavorable outcomes decreased by 21.5% (93 individuals) in the after-period (Table 2).

**Table 2:** Clinical characteristics of the study population.

Variable	Total (n = 26,842)	Before (n = 17,105)	After (n = 9,737)	P-value
Arrival type at ED				<0.001
Primary visit to ED	24,599 (91.6)	15,614 (91.3)	8,985 (92.3)	
Inter-hospital transfer	2,205 (8.2)	1,478 (8.6)	727 (7.5)	
Visiting ED via OPD	38 (0.1)	13 (0.1)	25 (0.3)	
Heart rate (bpm) ^a^				<0.001
60–100	22,898 (85.7)	14,695 (86.2)	8,203 (84.9)	
< 60	817 (3.1)	450 (2.6)	367 (3.8)	
> 100	3,005 (11.2)	1,910 (11.2)	1,095 (11.3)	
SBP (mmHg) ^b^				0.004
≥ 90	25,904 (97.4)	16,624 (97.6)	9,366 (97.1)	
< 90	687 (2.6)	403 (2.4)	284 (2.9)	
RR (breaths/min) ^c^				<0.001
12–20	24,702 (92.6)	15,677 (92.1)	9,025 (93.5)	
< 12	343 (1.3)	196 (1.2)	147 (1.5)	
> 20	1,629 (6.1)	1,152 (6.8)	477 (4.9)	
BT (°C) ^d^				<0.001
35.5–37.5	25,398 (94.9)	16,351 (95.9)	9,047 (93.2)	
< 35.5	600 (2.2)	328 (1.9)	272 (2.8)	
> 37.5	760 (2.8)	372 (2.2)	388 (4.0)	
Level of consciousness				0.143
Alert	25,205 (93.9)	16,078 (94.0)	9,127 (93.7)	
Verbal	607 (2.3)	396 (2.3)	211 (2.2)	
Pain	483 (1.8)	307 (1.8)	176 (1.8)	
Unresponsive	547 (2.0)	324 (1.9)	223 (2.3)	
Emergency surgery				0.142
No	26,103 (97.2)	16,653 (97.4)	9,450 (97.1)	
Yes	739 (2.8)	452 (2.6)	287 (2.9)	
ICU admission				0.016
No	24,907 (92.8)	15,921 (93.1)	8,986 (92.3)	
Yes	1,935 (7.2)	1,184 (6.9)	751 (7.7)	
Unfavorable outcome				<0.001
No	26,071 (97.1)	16,673 (97.5)	9,398 (96.5)	
Yes	771 (2.9)	432 (2.5)	339 (3.5)	

Presented as number (%)

^a^122 missing data point,

^b^251 missing data point,

^c^168 missing data point,

^d^84 missing data point. SBP, systolic blood pressure; RR, respiratory rate; BT, body temperature; ICU, intensive care unit.

### Severity and prognosis of the pedestrian TA patients according to age

Table 3 shows the severity and prognosis of the study participants according to age. The number of P-TA patients decreased in all severity groups, but the reduction trend differed according to severity. For cases of moderate and mild severity (urgent, less urgent, and non-urgent KTAS groups), the number decreased from 15,630–8,751—a 44.0% reduction (6,789 individuals). Meanwhile, the number of severe cases (resuscitation and emergent KTAS groups) decreased from 1,475–986—a 33.2% reduction (489 individuals). Furthermore, the reduction rate was lower in the severe group than in the mild group. For patients aged 80 years and older, the number of patients decreased by 27 (6.7%) at the urgent level and 7 (6.5%) at the emergency level. Moreover, in the 70s age group, there was an increase in two patients (2.7%) at the resuscitation level. Regarding emergency surgery and ICU admission, a decrease was observed in only 12 (22.2%) and 28 patients (18.5%), respectively, among those aged 80 and above. Additionally, concerning unfavorable outcomes, a decrease was noted in only 12 patients (13.3%) aged 80 years and older, and there was an increase of 10 patients (9.7%) in the 70s. This suggests that lowering the maximum speed limit on roads has a relatively limited effect on the reduction rates of severely ill and geriatric P-TA patients.

**Table 3:** Distribution of KTAS levels and outcomes in the study population by age.

Variable	20s	30s	40s	50s	60s	70s	≥80	Total
KTAS level								
Resuscitation								
Before	30 (7.1)	32 (7.5)	38 (8.9)	108 (25.4)	89 (20.9)	74 (17.4)	54 (12.7)	425
After	19 (6.4)	12 (4.1)	30 (10.2)	49 (16.6)	65 (22.0)	76 (25.8)	44 (14.9)	295
Emergent								
Before	89 (8.5)	88 (8.4)	124 (11.8)	207 (19.7)	215 (20.5)	220 (21.0)	107 (10.2)	1,050
After	44 (6.4)	58 (8.4)	54 (7.8)	121 (17.5)	148 (21.4)	166 (24.0)	100 (14.5)	691
Urgent								
Before	368 (9.2)	316 (7.9)	511 (12.7)	823 (20.5)	832 (20.7)	769 (19.1)	402 (10.0)	4,021
After	262 (9.1)	258 (8.9)	256 (8.9)	495 (17.2)	643 (22.3)	595 (20.6)	375 (13.0)	2,884
Less-urgent								
Before	1,749 (18.8)	1,201 (12.9)	1,237 (13.3)	1,869 (20.1)	1,543 (16.6)	1,223 (13.2)	461 (5.0)	9,283
After	933 (19.1)	588 (12.1)	609 (12.5)	842 (17.3)	956 (19.6)	637 (13.1)	313 (6.4)	4,878
Non-urgent								
Before	624 (26.8)	328 (14.1)	358 (15.4)	423 (18.2)	328 (14.1)	212 (9.1)	53 (2.3)	2,326
After	248 (25.1)	130 (13.1)	128 (12.9)	152 (15.4)	181 (18.3)	108 (10.9)	42 (4.2)	989
Emergency surgery, yes								
Before	28 (5.3)	39 (6.5)	46 (10.1)	92 (20.6)	109 (22.2)	84 (22.6)	54 (12.8)	452
After	10 (6.0)	20 (4.4)	24 (7.3)	58 (17.0)	68 (22.9)	65 (26.0)	42 (16.4)	287
ICU admission, yes								
Before	63 (6.2)	77 (8.6)	119 (10.2)	244 (20.4)	263 (24.1)	267 (18.6)	151 (11.9)	1,184
After	45 (3.5)	33 (7.0)	55 (8.4)	128 (20.2)	172 (23.7)	195 (22.6)	123 (14.6)	751
Unfavorable outcome, yes								
Before	23 (5.3)	16 (3.7)	29 (6.7)	83 (19.2)	88 (20.4)	103 (23.8)	90 (20.8)	432
After	16 (4.7)	10 (2.9)	20 (5.9)	39 (11.5)	63 (18.6)	113 (33.3)	78 (23.0)	339

Presented as number (%)

KTAS, Korean Triage and Acuity Scale; ICU, Intensive care unit

### Impact of lowering maximum speed limit of city roads on prognosis of the pedestrian TA patients

Table 4 presents the results of the univariable and multivariable logistic regression analyses of the medical prognosis of the study participants. In the multivariable logistic regression analysis, the risk of emergency surgery was not significantly different between the two periods (aOR, 1.130; 95% CI, 0.950–1.343). Nevertheless, the risk of ICU admission and unfavorable outcomes significantly increased in the after-period, despite policy implementation (aOR, 1.229; 95% CI, 1.085–1.392 for ICU admission; aOR, 1.344; 95% CI, 1.037–1.742 for unfavorable outcomes).

**Table 4:** Univariate and multivariate logistic regression analyses of the impact of Safe-Speed-5030 on the study population.

Variable	Univariable		Multivariable*	
**Crude OR**	**95% CI**	**aOR**	**95% CI**
Emergency surgery	1.119	0.963-1.300	1.130	0.950-1.343
ICU admission	1.124	1.022-1.236	1.229	1.085-1.392
Unfavorable outcome	1.392	1.205-1.609	1.344	1.037-1.742

*Adjusted for sex, age, region of ED, healthcare insurance type, time of accident, ED level, arrival type at ED, systolic blood pressure, heart rate, respiratory rate, body temperature, and level of consciousness.

OR, odds ratio; CI, confidence interval; aOR, adjusted odds ratio; ICU, intensive care unit; ED, emergency department.

## Discussion

To the best of our knowledge, this is the first nationwide study on the impact of lowering the maximum speed limit on city roads to improve the number and medical outcomes of P-TA patients in South Korea. The number of overall P-TA patients decreased significantly, as did that of patients with ED deaths, emergency surgeries, ICU admissions, and unfavorable outcomes. However, the reduction rate varied according to severity and age group, and the risk of ICU admission and unfavorable outcomes was higher in the after-period despite policy implementation.

As of 2020, South Korea had a TA death rate of 1.1 per 10,000 cars, which is the 4th highest among the 37 OECD countries. Additionally, the number of P-TA deaths among seniors aged 65 or older ranks first [16]. Considering the vulnerable nature of pedestrians exposed to the outside without safety equipment and domestic situations where aging is accelerating, this is an important issue that must be addressed urgently. Lowering vehicle speed is a considerable method for reducing TA occurrence and improving the medical outcomes of TA patients. Martin et al. reported that the risk of P-TA fatalities is lower when the vehicle speed is below 40 km/h and that the fatality rate doubles at 40 km/h and increases six-fold at 50 km/h, compared to a vehicle speed of 30 km/h [17]. Anderson et al. reported that reducing the maximum speed limit of vehicles from 60 km/h to 50 km/h could reduce the number of fatal pedestrian accidents by 13% [18]. Nightingale et al. reported that a city-wide 20 mph speed limit can decrease traffic speed without reducing traffic volume [19]. Conversely, a previous study reported that the number of TAs increased when the maximum road speed limits were low, and the severity increased when the speed limits were high [20]. In South Korea, there are no reports regarding maximum road speed limit reduction policies and P-TAs; however, speed bumps reduce vehicle speed and the number and severity of P-TA patients [21]. In this study, it was found that the total number of P-TA patients decreased by 43.1%; therefore, we believe that the Safe-speed-5030 policy had a positive effect.

In this study, although the number of P-TA patients decreased in all severity groups, the rate of decrease was lower among the geriatric population, and the reduction trend was lower in the severe group, especially in the severe geriatric group. Furthermore, in the 70s age group, the number of P-TA patients presented with the highest severity and unfavorable outcomes increased. These results indicate effect of the Safe-speed-5030 policy were varied across severity and age groups. Fridman et al. reported that lowering the maximum road speed limit from 40 to 30 km/h reduced the severity and incidence of accidents, although the difference was not statistically significant [22]. However, in geriatrics, cognitive decline can lead to a slower perception of dangerous situations and reduced reflexes, and decreased muscular power makes it difficult for them to perform swift evasive actions. Additionally, it is often challenging for geriatrics to perceive external risk factors owing to the decline in sensory functions, such as vision or hearing; thus, they are challenged to evade approaching high-speed vehicles. Therefore, it is possible that speed reduction was not sufficient for geriatric patients who are more vulnerable to severe injuries. Furthermore, sudden, rapidly approaching high-speed vehicles could be more difficult to avoid than low-speed vehicles, and these vehicles have a higher possibility of causing serious damage when crashing into pedestrians. Therefore, although this policy was effective in preventing relatively minor P-TAs, its impact on serious TAs caused by high-speed vehicles may have been limited. These factors may have contributed to the relatively low reduction rate in geriatric or severe P-TAs despite the lowering of the maximum road speed limit observed in this study. Therefore, a more effective policy that considers these factors is required.

Drivers with a history of traffic violations, such as speeding, negative perceptions of public regulations, or aggressive driving habits, are more likely to cause accidents. Conversely, drivers who adhere to speed limits may also be more inclined to obey other traffic laws, such as obeying signals [23,24]. Moreover, at lower speeds, drivers are better able to respond to sudden situations and have more time to evade potential accidents. Therefore, although stricter maximum speed limit policies were implemented, their impact may have been more significant for law-abiding drivers, while their effect on aggressive drivers may have been minimal. These aggressive drivers are likely to continue violating traffic laws and disregarding speed limits. As a result, there may have been a relatively greater reduction in minor TAs compared to severe TAs. In this study, the number of patients requiring emergency surgery, ICU admission, ED deaths, and unfavorable outcomes decreased in the after-period. Nevertheless, the reduction rate in the number of ED deaths and unfavorable outcomes was lower than the overall reduction rate. Moreover, the risk of emergency surgery was not statistically different between the two periods, and the risk of ICU admission and unfavorable outcomes was slightly higher in the post-period, despite lowering the maximum road speed limit. The prognosis of trauma patients is influenced by various factors such as patient severity, field management, the time it takes to arrive at a medical institution providing final treatment, such as emergency surgery after a TA, and medical resources available to provide final treatment. We presume that the reason for the difference in ICU admission and outcomes between the two periods in this study is that, although the overall number of patients decreased, the decrease ratio of severe patients was lower than that of minor patients, and severe patients had a higher possibility of intensive management and unfavorable outcomes. Thus, in addition to reducing the number of P-TA patients and increasing the availability of ED and ICU beds, it is necessary to implement other policies to improve outcomes for P-TA patients. This may include securing manpower for key emergency medical management and establishing a prehospital medical direction system for severe TA patients. This enables the provision of a more effective emergency medical service system for P-TA patients.

One important point is that the after-period of this study occurred under the unique circumstances of the COVID-19 pandemic, which may have impacted traffic density. Although there are reports of increased traffic density in some cities, most cities experienced an overall reduction in traffic density during the COVID-19 period [25,26]. A decrease in traffic density may reduce minor TAs, and the reduction in minor TA cases during the COVID-19 pandemic could have contributed to the overall decrease in P-TAs. On the other hand, while a short-term decrease in traffic density could potentially lead to an increase in severe casualties due to factors such as drivers’ propensity to speed [27]. This study targeted level-1 and level-2 EDs, which primarily handle moderate to severe casualties, rather than minor cases, regardless of special circumstances such as the COVID-19 pandemic. Additionally, the primary goal of the Safe Speed-5030 policy was to reduce the number of severe casualties. Despite the potential for an increase in severe injuries due to reduced traffic density from the COVID-19 pandemic, the number of P-TA patients decreased across all severity groups, including serious casualties, following the implementation of the policy in this study. Therefore, we believe that the Safe-Speed-5030 policy was somewhat effective in achieving its primary goal of reducing severe P-TA casualties. However, the occurrence of P-TA is influenced by various factors in a complex manner, and changes in traffic density may be one of these factors. Thus, future studies that consider the interaction between these factors are necessary.

One key aspect to consider is the controversy and dissatisfaction expressed by citizens regarding the policy. Owing to driver dissatisfaction caused by the uniform enforcement of the Saft-Speed-5030 policy, some local authorities have recently considered reverting to the previous maximum road speed limit [28]. However, Hussain et al. proposed speed limits of 30–40 km/h in pedestrian-dense areas and reported that such policies are utilized in countries with low P-TA rates [29]. Furthermore, there was a considerable reduction in the number of P-TAs after the implementation of the Safe-speed-5030 policy. Considering these factors, it might be appropriate to consider improved methods, such as selective speed limits in TA-prone or pedestrian-dense areas, as well as implementing speed limits based on pedestrian flow over time, rather than abolishing the Safe-speed-5030 policy. Moreover, it is necessary to consider certain additional interventions: installing alternative facilities for crosswalks, including pedestrian bridges in areas with high pedestrian traffic; designing roads with consideration for geriatrics, such as extending the operating times of crosswalks; improving road lighting and pavement conditions [30,31] Complementary measures of the policy through continued evaluation and multifocal approach may strengthen its effectiveness and address any remaining concerns or controversies [32]. In this regard, this study provides valuable evidence supporting the effectiveness of the Safe-Speed-5030 policy in reducing the incidence and severity of P-TAs in South Korea. We believe in the importance of implementing policies that benefit the public, even in the face of social controversy. The findings of this study could lead to constructive discussions on the merits and potential modifications of the Safe-Speed-5030 policy. Additionally, this study can contribute to ongoing decision-making processes, ultimately enhancing road safety and protecting all road users, including pedestrians.

This study has several limitations. First, it was conducted using NEDIS data for P-TA patients from seven major cities in South Korea. However, public traffic policies may be influenced by various factors, such as traffic culture, the legal environment, and other socioeconomic conditions, which can vary across regions. Additionally, the severity of TAs can be affected by factors such as the types of accidents, causes, locations, weather conditions at the time, road type and condition, traffic volume, and kinetic factors, including impact speed and vehicle types. However, the NEDIS does not collect these data, and accurately compiling all the aforementioned information was practically impossible. Therefore, these factors should be considered when interpreting the results, and we believe that more comprehensive research, including international studies, will be needed. Second, the study participants were P-TA patients managed at level-1 and level-2 emergency medical centers in South Korea. Thus, it is possible that minor TA patients visited level-3 emergency centers or general clinics were excluded. This should be considered when interpreting the results. Nonetheless, TA patients suspected of having severe trauma in an accidental area are generally transferred to level-1 or level-2 emergency centers by public EMS. Moreover, if patients who visited a level-3 emergency center or general clinic had a major injury, they were transferred to an advanced-level EDs. Hence, we believe that the effect of this matter on investigating the impact of the Safe-speed-5030 policy for major P-TA patients was limited. Finally, given that this policy was implemented on urban roads, caution should be exercised when generalizing the findings to other types of roads, such as rural roads or highways. Further research that considers these factors will be needed.

## Conclusions

After the implementation of the Safe-Speed-5030 policy, the number of patients with P-TA decreased dramatically; however, the positive effect varied by severity and age, and the prognosis worsened. Considering these aspects, it is believed that the Safe Speed-5030 policy has shown some positive effects on P-TAs. Therefore, it is more appropriate to apply revised policies rather than completely scrap them, and additional measures are required to improve the prognosis of patients with severe and geriatric P-TAs.
